# Effectivity of zinc oxide-turmeric extract dressing in stimulating the reepithelization phase of wound healing

**DOI:** 10.14202/vetworld.2020.2221-2225

**Published:** 2020-10-27

**Authors:** Asti Meizarini, Aryati Aryati, Devi Rianti, Wibi Riawan, Astari Puteri

**Affiliations:** 1 Department of Dental Material, Faculty of Dental Medicine, Universitas Airlangga, Surabaya, Indonesia; 2 Department of Clinical Pathology, Faculty of Medicine, Universitas Airlangga, Surabaya, Indonesia; 3 Department of Biochemistry and Molecular Biology, Faculty of Medicine, Universitas Brawijaya, Malang, Indonesia; 4 Department of Oral and Maxillofacial Pathology, Faculty of Dental Medicine, Universitas Airlangga, Surabaya, Indonesia

**Keywords:** cytokeratin 14, epithelial cadherin, epidermal growth factor receptor, turmeric extract, wound dressing

## Abstract

**Background and Aim:**

Reepithelialization can be described as the resurfacing of a wound with new epithelium in the process of healing, with the overlapping step from keratinocyte migration and proliferation to the tissue contraction. Zinc oxide-turmeric extract dressing has been proven to have anti-inflammatory properties, but its effectivity in the reepithelialization process is still unknown. This study aimed to determine the effect of a wound dressing consisting of zinc oxide and turmeric extract on wound reepithelialization by assessing the expression of cytokeratin 14 (CK14), epidermal growth factor receptor (EGFR), and epithelial cadherin (E-cadherin).

**Materials and Methods:**

A total of 40 Wistar rats were randomized into four control and four treatment groups (n=5 per group). On day 1, a square-shaped full-thickness skin excision measuring 6×6 mm in size was created in the dorsal thoracic area of the rats, and the wounds were either dressed with a combination of zinc oxide and turmeric extract in the treatment groups or left undressed in the control groups. Then, the rats were sequentially sacrificed on days 3, 5, 7, and 14 to obtain subepithelial excision samples, which were subsequently subjected to immunohistochemistry analysis for the expression of CK14, EGFR, and E-cadherin to ascertain wound reepithelization. The data were tabulated and analyzed using a one-way analysis of variance and least significant difference test.

**Results:**

The highest expression levels of CK14, EGFR, and E-cadherin were observed on days 7 and 14 in the treatment and control groups, respectively. While the expression levels of these markers on day 7 were found to be significantly higher in the treatment than the control groups, no significant difference in the expression levels on day 14 was detected between the control and treatment groups (p<0.05).

**Conclusion:**

A wound dressing consisting of zinc oxide and turmeric extract can help accelerate reepithelization in the wound healing process.

## Introduction

Epithelialization, which is an essential component of wound healing, is often used as a success parameter. Reepithelialization can be described as the resurfacing of a wound with new epithelium, which starts approximately 16-24 h after injury (i.e., precisely during the proliferation phase). The wound healing process involves blood clot formation, inflammation, reepithelialization by keratinocyte migration and proliferation, granulation tissue formation, neovascularization, and tissue contraction, with these steps overlapping largely with one another [1].

Keratinocytes, the major cellular component of the epidermis, are crucial not only for barrier maintenance but also for its restoration on injury through a process known as epithelialization [2]. They are primarily composed of intermediate filament (IF)-forming proteins typical of epithelial cells, called keratins (Ks) or cytokeratins (CKs), which are essential for healthy tissue structure [3]. While epithelial tissues express different pairs of Ks depending on the cell type, all stratified squamous epithelia express K5/K14 in the basal layer [4]. Numerous factors control keratinocyte differentiation, such as epidermal growth factor (EGF), which exerts a mitogenic effect on basal cells through interaction with EGF receptors (EGFRs) [5]. After tissue injury, EGFR is strongly upregulated in keratinocytes at the wound edge and remains present in the hypertrophic proliferating area of the epithelium until the proliferative phase ceases. Studies using cultured keratinocytes, as well as animal wound healing models, have proven that EGFR signaling might promote reepithelialization by at least three distinct mechanisms: (1) Collaborating with integrins to activate the mitogen-activated protein kinase pathway [6]; (2) disintegrating desmosomal and hemidesmosomal adhesions and remodeling cell-cell and cell-substratum contacts to allow the migration of keratinocytes [2]; and (3) stimulating the expression of integrins, extracellular proteases, and matrix molecules that facilitate reepithelialization [7]. Moreover, EGFR activation has been linked to intercellular junctions and actomyosin organization [8]. It has been shown that EGFR itself is directly regulated by epithelial cadherin (E-cadherin), which is a member of the cadherin family and plays a key role in cell-cell adhesion and signaling [8,9]. Staining for E-cadherin has confirmed the presence of E-cadherin-containing adherens junctions at the sites of cell-cell contacts in all epidermal layers, with no apparent enrichment in the granular layer, where tight junctions are formed [8].

 A wound dressing comprised zinc oxide combined with turmeric extract has been proven to have anti-inflammatory properties, therefore contributing to the healing of skin excisional wounds in rats [10]. A previous study showed this combination to be capable of accelerating the inflammatory stages of the wound healing process by increasing the number of macrophages earlier than normal and lowering toll-like receptor 2, nuclear factor-kappa B, tumor necrosis factor-alpha, and cyclooxygenase 2 [10,11]. Nevertheless, its effectivity in the reepithelialization process is still unknown.

This study aimed to investigate the effect of a wound dressing consisting of zinc oxide and turmeric extract on wound reepithelialization through the expression of CK14, EGFR, and E-cadherin.

## Materials and Methods

### Ethical approval

This research was a post-test only control group design study. The methods were reviewed and approved by the Ethical Committee Board No. 139/HRECC.FODM/VIII/2017.

### Study period and location

****This study was conducted from July to September 2018 at Biochemistry and Molecular Biology Laboratorium, Faculty of Medicine, Universitas Brawijaya, Malang, Indonesia.

### Wound dressing preparation

In brief, 0.3 g of zinc oxide powder (Merck KGaA, Darmstadt, Germany) and 0.3 g of turmeric liquid extract (Balai Materia Medica, Batu, Indonesia) were mixed together on a mixing pad with a stainless-steel spatula for 60 s before reaching a homogenous consistency. The prepared wound dressing was then applied to the wounds.

### Animals and experimental design

**Figure-1:**
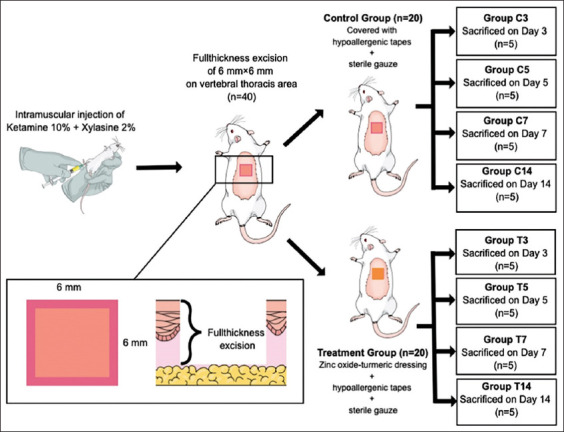
The experimental design in the animal.

In total, 40 male Wistar rats (*Rattus norvegicus*) weighing 200-300 g were obtained from Wistar Farm (Malang, Indonesia) and were allowed to adapt to the new environment for a week before being divided into four control (namely, C3, C5, C7, and C14) and four treatment groups (namely, T3, T5, T7, and T14) of five animals each. On day 1, a square-shaped full-thickness skin excision measuring 6 × 6 mm in size was created in the dorsal thoracic area of the rats (Figure-1), and the wounds were either dressed with a combination of zinc oxide and turmeric extract in the treatment groups or left undressed in the control groups. All the wounds were then covered with hypoallergenic tapes (BSN Medical, Hamburg, Deutschland) and sterile gauze. Afterward, the rats were sequentially sacrificed on days 3 (C3, T3), 5 (C5, T5), 7 (C7, T7), and 14 (C14, T14) to obtain subepithelial excision samples, which were subsequently analyzed immunohistochemically for the expression of CK14, EGFR, and E-cadherin to examine wound reepithelization.

### Immunohistochemistry staining

The samples were fixed in 10% neutral buffered formalin for 48 h, dehydrated with ethanol and embedded in paraffin blocks. Sections of 4 μm thickness were cut from the paraffin blocks and mounted on slides. Immunohistochemical staining for CK14, EGFR, and E-cadherin was performed using the following mouse monoclonal antibodies, respectively: CK14 (clone LL001; Santa Cruz Biotechnology, Dallas, USA), EGFR sc-373746 (clone A-10; Santa Cruz Biotechnology, Dallas, USA), and E-cadherin sc-8426 (clone G-10; Santa Cruz Biotechnology, Dallas, USA). The secondary antibody used was CRF Anti-Polyvalent HRP Polymer (anti-mouse, anti-rabbit; ScyTek Laboratories, Logan-Utah, USA).

The expression of CK14, EGFR, and E-cadherin was observed under a light microscope (Nikon Eclipse E 100, Tokyo, Japan) at 1000× by two different individuals, who separately counted 20 fields of view, and the average was used for each slide. Images were captured using a Sony ILCE A7 camera (Sony, Tokyo, Japan).

### Statistical analysis

Data collected were tabulated and analyzed using a one-way analysis of variance and least significant difference test in SPSS version 21 (IBM, New York, USA).

## Results

**Figure-2:**
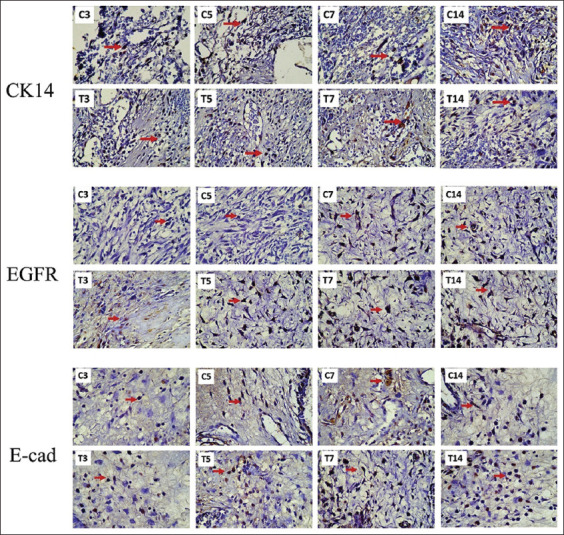
The expression of cytokeratin 14, epidermal growth factor receptor, and E-cadherin in the basal layer at 1000× indicated by the red arrow in both the control (C3, C5, C7, and C14) and treatment (T3, T5, T7, and T14) groups.

**Figure-3:**
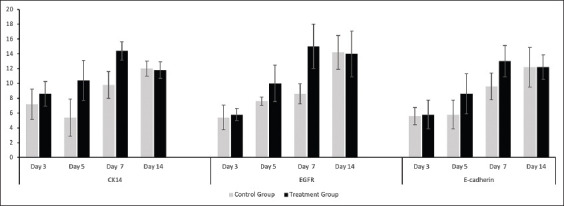
Mean and standard deviation of cytokeratin 14, epidermal growth factor receptor, and E-cadherin expression on the control and treatment groups on day 3, 5, 7, and 14.

Representative images of the immunohistochemical expression of CK14, EGFR, and E-cadherin in the basal layer are shown in Figure-2. No significant difference was found between the control and treatment groups regarding the expression levels of these markers on day 3. Yet, the expression levels in the treated rats rose on day 5, peaked on day 7, and then decreased on day 14 (Figure-3). On the contrary, the expression levels in the control rats showed an increasing trend until day 14, except for CK-14, which had a decrease on day 5 before having an increasing trend until day 14.

The highest expression levels of CK14, EGFR, and E-cadherin were observed on days 7 and 14 in the treatment and control groups, respectively. Although the expression levels of these markers on day 7 appeared to be significantly higher in the treatment than the control groups (p<0.05), no significant difference in the expression levels on day 14 was detected between the control and treatment groups.

## Discussion

In general, the expression levels of CK14, EGFR, and E-cadherin in the treated rats increased gradually to reach a peak on day 7 before declining on day 14. In the control rats, however, the expression levels increased slowly until they peaked on day 14. These trends indicate that reepithelialization was completed earlier in the rats dressed with the combination of zinc oxide and turmeric extract compared to the untreated controls. This finding is in accordance with a previous study, wherein the topical application of curcumin products was shown to result in quicker reepithelialization, collagen deposition, and wound reduction [12].

It has been shown that once the skin barrier is disrupted through acute injury, neutrophils, monocytes, and macrophages are recruited to the site of injury, followed by activation of keratinocytes, which – in the basal layer – are proliferative and characterized by an infrastructure composed of K-containing IFs (e.g., K5 and K14) [2,13]. Furthermore, an *in vitro* study documented keratinocyte migration 1 day after wounding; it also demonstrated that, within 4-5 days, keratinocytes covered the whole wound area and formed a neoepidermis, which, in turn, began to thicken and form a multilayered epithelium from days 4 to 10 [14].

According to the results of immunohistochemical analysis, the expression of CK14 in the control rats rose significantly on day 7 and continued to increase until day 14 due to the ongoing keratinocyte proliferation necessary for neoepidermis formation and thickening, thus contributing to the differentiation process. Only basal keratinocytes have the ability to proliferate, whereas terminally differentiated keratinocytes in the suprabasal layer have lost this ability [1]. The increased expression of CK14 in the rats dressed with zinc oxide and turmeric extract occurred even faster, with its level peaking on day 7 and slowly declining thereafter. This result suggests that the topical application of zinc oxide and turmeric extracts to wounds might accelerate keratinocyte migration to the site of injury and consequently keratinocyte proliferation in the basal layer to form a thick neoepidermis. As these cells move upward to the skin surface, they start to differentiate and lose their proliferation potential, leading to a gradual reduction in the levels of K5/K14 [4]. It is noteworthy that the process of differentiation involves switching from the synthesis of K5 and K14 in the basal layer to K1 and K10 in suprabasal layers [2].

EGF and transforming growth factor-α, which are among numerous factors controlling keratinocyte differentiation, have been proven to exert a mitogenic effect on basal cells through interaction with EGFRs on the basal cells of the oral mucosa [5]. Besides, it has been demonstrated that after tissue injury, EGFR is strongly upregulated in keratinocytes at the wound edge until the proliferative phase ceases [15]. In a previous study, although EGFR was found to be restricted to the basal area during early stages of epidermal morphogenesis, staining of control tissue sections revealed localization of EGFR not only in the basal layer but also in suprabasal layers [8]. The expression of EGFR in the control rats exhibited an increase on day 5, was relatively stable on day 7, and then escalated significantly on day 14, indicating that the proliferation of CK14 and EGFR which needed for epithelial formation, were still in progress on day 14. On the other hand, the expression of EGFR in the rats dressed with zinc oxide and turmeric extract increased on day 5, reached its peak on day 7, and diminished slightly on day 14. Since EGFR expression has been associated with keratinocyte proliferation [15] and CK14 expression, the dressing implemented herein showed the ability to induce epithelial cell proliferation and junction formation more quickly.

E-cadherin directly regulates EGFR [8]. The expression of E-cadherin in the basal layer of the control rats significantly increased on day 7 and followed a progressive upward trend until day 14. With respect to the treatment groups, however, the increase in E-cadherin expression began earlier on day 5 and reached its peak on day 7, followed by a modest decline on day 14. This implies that the zinc oxide-turmeric extract dressing could help the formation of adherens junctions happen faster. Hence, cadherin-dependent regulation of suprabasal junctional EGFR is deemed essential not only for the establishment of epithelial barrier function but also for regeneration of the barrier and restoration of homeostasis on barrier disruption [8].

## Conclusion

Zinc oxide-turmeric extract dressings can accelerate the formation of keratinocytes (in the basal layer), epithelium, and junctional epithelium, which, in turn, lead to faster reepithelialization as epidermal homeostasis.

## Authors’ Contributions

AM, AA, and DR were responsible for the design of the experimental study. AM, AP, DR, and WR performed the experiment and immunohistochemistry. WR performed the statistical analysis. AM, AP, and DR prepared the manuscript. AA and WR revised the manuscript critically. All authors read and approved the final manuscript.
